# Abutment conditions in faulty prosthesis among Indians

**DOI:** 10.6026/973206300171101

**Published:** 2021-12-31

**Authors:** S Harrita, V Suresh, P Senthil Murugan

**Affiliations:** 1 Saveetha Dental College and Hospitals, Saveetha Institute of Medical and Technical Sciences, Saveetha University, Chennai, India

**Keywords:** Faulty prosthesis, Quacks, unqualified dentists

## Abstract

A faulty prosthesis can cause damage more than relief. Poor people who cannot afford specialty treatment prefer to go to unregistered dental practitioners who are less expensive. Therefore, it is of interest to record the presence and type of old faulty prosthesis and its effect on surrounding structures. In this study 983 case sheets were reviewed from the record management system at the Saveetha Dental College, India using keyword search. Results show that 33% of faulty prosthesis leads to periodontally compromised abutments, 26% to decay of abutment tooth, 20% to gingival inflammation, 13% to denture stomatitis, 6.6% to non-vital abutment tooth. Faulty prosthesis damages abutment tooth and the surrounding structure of oral mucosa. Thus, damage to the periodontium in fixed prosthesis is common and prevalent. Hence, faulty dental prosthesis construction should be discouraged through awareness programme.

## Background:

A dental prosthesis is a protheis used to restore residual ridge defects, missing teeth, missing structure of teeth and associated structures around it [1]. A fixed dental prosthesis failure maybe biological mechanical or aesthetic, it could cause discomfort caries, pulpal injury, periodontal breakdown, occlusal problems, tooth perforation, tooth fracture, loosening or dislodgement prosthetic fracture, occlusal wear or perforation [1,2]. In separate studies, caries and loss of retention were identified as the major events complicating FDP performance [3]. Faulty prosthesis can lead to harmful effects on the residual ridges; these serious effects are further compromised if the patient is systemically compromised [4]. The quackery practice is harmful in medicine, dentistry that creates a disillusionment of the professionals and professional ethics [5]. There is a lack of data and literature regarding the number of quacks in India or their treatments or their mismanagements. Also no solid data are available regarding the number of practicing quacks or unqualified dental practitioners by virtue of their profession since many years [6]. Quacks are those who have observed and self learned a few techniques of dentistry either by assisting dental surgeons or inherited it from their families and adopted it as a profession [7]. Unwary patients hoping for a quick and easy solution to their dental problem frequently end up with botched procedures that are not only painful but also destructive. These untrained so-called professionals can frequently cause more traumas than good, and in some cases, irreparable damage. The common malpractice in India includes use of wires to stabilize the tooth or denture with the support of adjacent teeth, which just damage the remaining healthy teeth. This procedure can be traumatic to the patients as they can lead to excess bone loss and adjacent tooth loss. Another common malpractice is replacement of a missing tooth with artificial teeth with auto polymer resin directly in the mouth. The cold cure acrylic used for this purpose does not completely cure and can cause damage to the underlying gums and leads to bone restoration on adjacent teeth, and it is also a known carcinogenic material with high monomer content [8] to identify faulty prosthesis and evaluate the condition of the abutment teeth or the oral structures adjacent or supported by it.

## Materials & Methods:

The retrospective analysis was an institutional study from the already existing patients data. Two examiners were involved in this study. The data was collected from the RDBMS database at the Saveetha Dental College, India. The database stores records of patients with their intra-oral and extra-oral photographs (taken with consent of the patients), their demographic details, personal history, medical history and the Dental findings. The data was collected for nine months from June 2019 to March 2020 from the internal database. We used keywords such as faulty prosthesis, damaged prosthesis, ligated prosthesis, faulty acrylic, ligated acrylic, removable prosthesis faulty to retrieve the required data. The search identified 34 cases and further refinement selected 12 case sheets with the exact match for the analysis. Collected data was verified with photographs obtained from the digital documentation. The inclusion criteria for search included faulty prosthesis and damaged prosthesis. Data was imported to the SPSS 23.0 software for statistical analysis. Independent variables include age, gender, and faulty prosthesis. Both descriptive and inferential statistics were done. Frequency distribution for age and gender was completed. Chi square test was completed to find the association at a significance level of 0.05.

## Results and Discussion:

**Figure 1:**
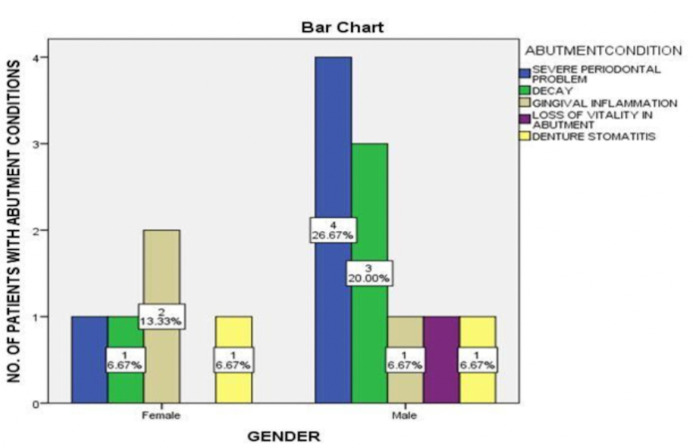
The graph shows the association between gender and abutment condition. Periodontally compromised abutments were more common in males than females (blue). However, this was not statistically significant from Pearson's Chi square analysis showing a p value of 0.596 (p>0.05)

**Figure 2:**
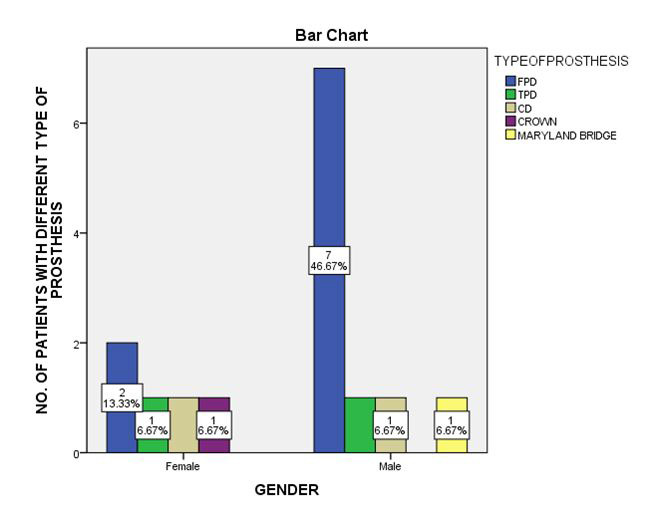
The graph shows correlation between gender and type of faulty prosthesis type. FPDs are the most common faulty prosthesis in both the genders (blue). The Pearson's chi-square analysis shows a p value of 0.478 (p>0.05).

**Figure 3:**
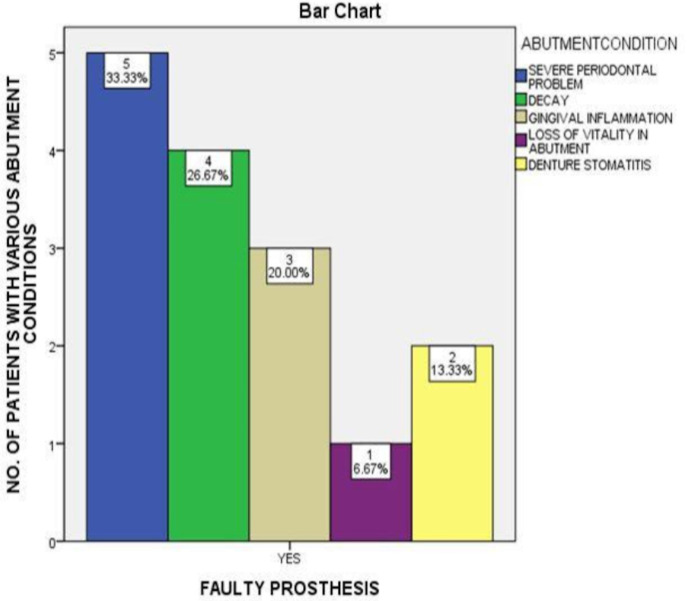
The graph shows association between faulty prosthesis and abutment condition. X-axis shows the presence of faulty prosthesis and y-axis shows count of patients with various abutment conditions. It is interfered that periodontally compromised abutments is the most common abutment condition in patients with faulty prosthesis (blue). The Pearson's chi-square analysis shows a p value of 0.088 (p>0.05).

**Figure 4:**
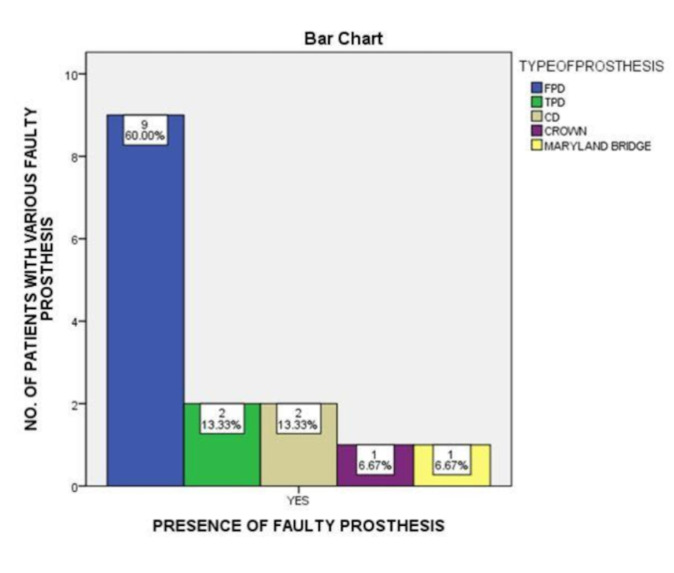
The graph shows the presence of faulty prosthesis with type of faults. The X-axis shows the presence of faulty prosthesis and the Y-axis shows the count for faulty prosthesis. FPDs were the most faulty prosthesis in this study (60%) as shown using blue.

The collected data was imported in SPSS software version 23 for chi-square test. Data analysis shows that 26.6% male and 6.6% female patients had periodontally compromised abutments, 20% male and 6.6% female patients had decay in their abutments, 6.6% male and 13.3% female patients had gingival inflammation in their abutments. Data also shows that 6.6% male had non-vital abutments. However, 6.6% male and 6.6% female patients had denture stomatitis. Nevertheless, this was not statistically significant (p>0.05). Thus, there is no significant association between gender and condition of abutment teeth (Figure 1). Association between gender and faulty prosthesis shows that FPDs were the most common faulty prosthesis in both genders (Figure 2). Nevertheless this was not statistically significant (p>0.05). Association between faulty prosthesis and abutment tooth condition shows that 33% of patients with faulty prosthesis had periodontally compromised abutment teeth, 26% had decay in the abutment teeth, 20% had gingival inflammation surrounding their abutment teeth, 6.6% had non-vital abutment teeth with FPDs and 13% had denture stomatitis. However, the p value was not statistically significant (>0.05). Thus, there is no significant association between the presence of faulty prosthesis and condition of abutment teeth (Figure 3). Nonetheless, the most common prosthesis at fault was FPDs (Figure 4). Pramod et al. suggests poorly designed prosthesis risks the oral health by making the oral site susceptible to mucosal ulcers leading to the necrosis of mucosa [9]. Chauhan et al. showed that the quack had adhered the denture with the adjacent natural tooth with the help of self-cure acrylic [10]. Such prosthesis caused damage to the surrounding structures in the oral cavity. There is no known data on quackery practice whereas there are evidence-based practices available on FPDs. Clinical studies indicate that ceramic chipping is common among FPDs. This results in technical complication where criteria for the success or failure of prosthesis are warranted [11, 12,13]. Removable prosthesis wired to the adjacent tooth or acrylic blocks directly placed in an edentulous area is advisable. Clinical findings with faulty prosthesis (denture stomatitis or inflammation of oral mucosa on the palatal aspect or on the gingival aspect directly) in directly contact with the denture baseare common [14]. Different factors like traumatic occlusion, poor oral hygiene and microbial growth are cause damage to abutment tooth [15]. Numerous clinical trials [16-23] and questionnaire based studies [24- 30] over the past 5 years point in these directions for dental practice.

## Conclusion:

Data shows that FPDs were the most faulty prosthesis followed by TPDs and CDs. Faulty prosthesis causes damage to the abutment tooth and the surrounding structure like the periodontium or oral mucosa. Thus, ill oral health caused by faulty prosthesis and quackery could be controlled by awareness.

## Author contribution:

All the authors contributed equally for the research.
